# Assessment of the diagnostic accuracy of clinical signs in feline diffuse iris melanoma

**DOI:** 10.3389/fvets.2026.1795617

**Published:** 2026-03-23

**Authors:** Joschka Spornberger, Petr Soukup, Ingrid Allgoewer

**Affiliations:** Animal Eye Practice, Berlin, Germany

**Keywords:** anterior uveal neoplasia, diagnostic accuracy, iris pigmentation, multivariate analysis, univariate analysis

## Abstract

**Objective:**

Retrospective evaluation of whether various clinical parameters and their combination led to an accurate diagnosis of diffuse iris melanoma in cats (FDIM), facilitating the veterinarian’s decision to enucleate the eye.

**Procedures:**

Medical records (2019–2024) of cats that underwent enucleation due to pigmentary iris lesions were reviewed. Eyes with a pathologically confirmed diagnosis (iris melanosis, FDIM) were included. Clinical signs were examined for their predictive value for FDIM using univariate and multivariate logistic regression.

**Results:**

The most common breeds were Domestic Shorthair (42/67) and British Shorthair (13/67). The average age was 10.2 ± 3.69 years. With each additional year of life, the probability of FDIM increased by 1.3x. Females had a higher risk of FDIM than males (24/27 female vs. 34/40 male, multivariate OR = 1.93). Univariate analysis revealed glaucoma, iris thickening and pigment dispersion (anterior chamber and lens capsule) had the highest OR. The multivariate analysis model (44 cats) showed the strongest positive effect for iris thickening (OR = 6.91) and pigment dispersion (OR = 4.87). Due to the small sample size, results were not statistically significant. Fifty-eight of 67 eyes were enucleated at veterinarian’s discretion, and 9 at the owner’s request. Of those eyes, FDIM was confirmed in 53 of 58 and 5 of 9, respectively (sensitivity 91%, specificity 44%).

**Conclusion:**

Based on clinical evaluation, the accuracy of the clinical diagnosis was correct in 85.07% of all cats with high sensitivity and low specificity. The strongest predictor of a correct clinical diagnosis of FDIM was iris thickening.

## 1 Introduction

**Figure 1:**
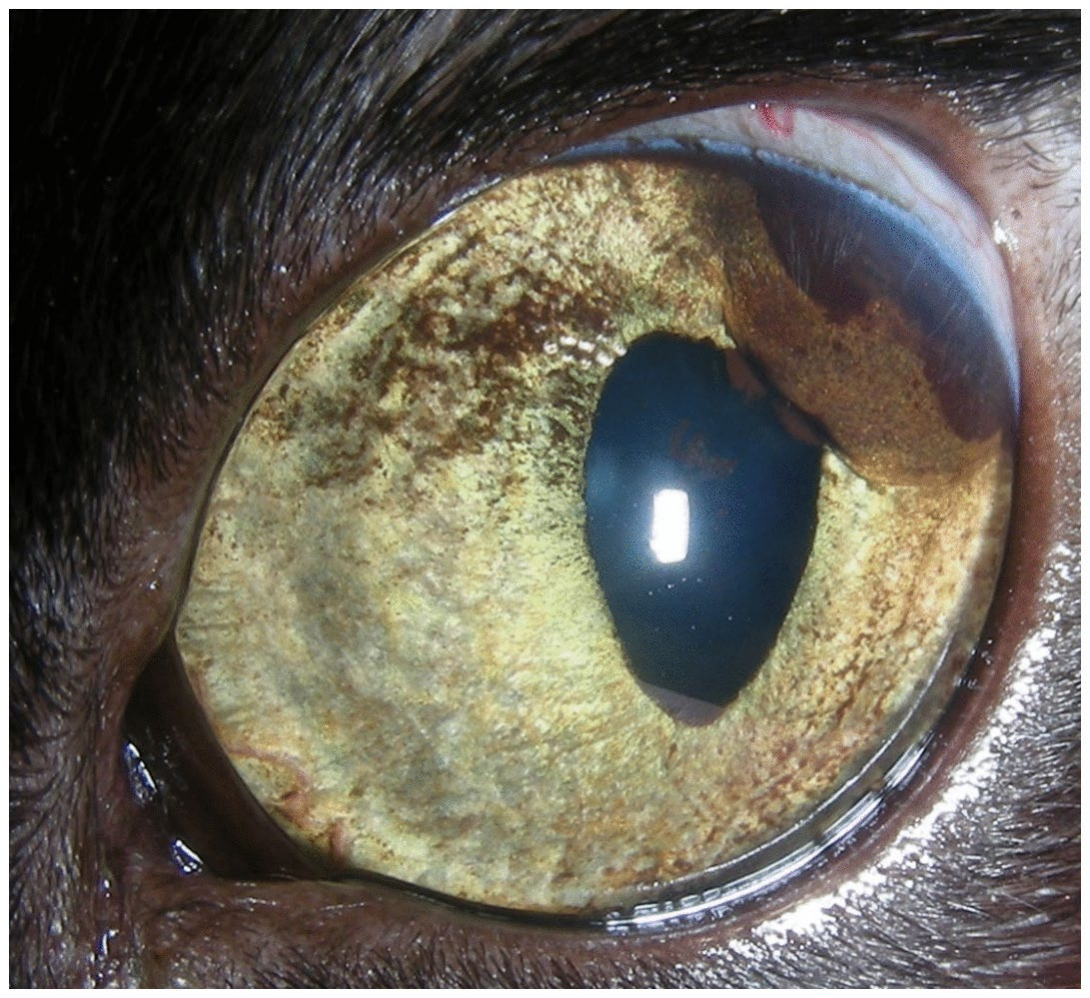
Close-up of a cat’s eye with starting iris pigmentation consistent with FDIM. Notable clinical features include nodular, raised, dark brown pigmentation in the temporal region of the iris, diffuse pigmentation of the anterior surface of the iris, partial dyscoria, posterior synechia, and pigment dispersion visible on the anterior lens capsule. The histopathological diagnosis of this eye was FDIM.

Feline diffuse iris melanoma (FDIM) is the most common primary intraocular neoplasm in cats, with variable progression (1, 2). The clinical signs of FDIM vary and range from increased pigmentation of the iris to advanced signs such as iris thickening, pigment dispersion in the anterior chamber or on the anterior lens capsule, dyscoria, and secondary glaucoma (2) (Figure 1).

Benign iris melanosis can manifest with clinical features that are indistinct from those of early-stage FDIM, making it difficult to distinguish between the two disease entities (2). Histopathologic evaluation of enucleated globes remains the gold standard for distinguishing between iris melanosis and FDIM (2). Iris cysts are yet another differential diagnosis for pigmented iris lesions. In the human literature, a comprehensive study of 200 cases of potential iris melanomas found that 76% of these were classified as pseudomelanomas (3). Of these pseudomelanomas, 39% were iris cysts and 31% were iris nevi (3). In 2017, Fragola et al. (4) reported a case series of 14 enucleated feline eyes due to suspected FDIM or “tumor” that contained only iris cysts. Some of these cysts exhibited clinical behavior similar to FDIM, such as dyscoria and progressive enlargement over time, which further complicated the assessment (4).

In clinical practice, veterinary ophthalmologists often decide whether enucleation is recommended based solely on the clinical appearance and progression of ocular signs. The owner’s reluctance to perform enucleation without prior histological confirmation can lead to a delay in treatment and thus to an increased risk of metastasis (5). Conversely, unnecessary enucleation due to misdiagnosis is undesirable for ethical reasons. Although several studies have described the histopathological and immunohistochemical features of FDIM (5–13), no peer-reviewed scientific literature has investigated the predictive value of clinical signs for distinguishing between FDIM and benign diseases. Fuchs et al. have sought to standardize the assessment of pigmented iris lesions. A stage-based grading scheme was proposed, whereby the progression of the disease is described by the increasing involvement of the iris with stromal thickening, pigment dispersion, dyscoria, uveitis, and secondary glaucoma (14). In the field of human ophthalmology, a predictive model for iris melanoma has been developed. This model identifies independent clinical predictors, including tumor height, the largest basal diameter, pupillary distortion (ectropion uveae or corectopia), peripheral extension, pigment dispersion, and vascularity. These findings emphasize the importance of the clinical examination in the diagnosis of iris melanoma (15). In veterinary patients, iris biopsy has been described as a minimally invasive procedure with low complication rates (16, 17). However, the diagnostic concordance of iris biopsy with globe histopathology is variable and influenced by sample quality and disease stage (18).

Understanding the predictive value of clinical findings such as iris thickening, pigment dispersion, or elevated intraocular pressure could significantly improve clinical decision-making. Therefore, the aim of this study was to retrospectively investigate whether selected clinical signs and their combinations led to an accurate diagnosis of FDIM. In addition, the potential predictive value of these parameters would be investigated.

## 2 Materials and methods

The medical records of cats that underwent enucleation between January 2019 and November 2024 at the Animal Eye Practice Dr. Allgoewer (Berlin, Germany), were retrospectively evaluated.

All cats participating in the study underwent a complete ophthalmic examination by a board-certified veterinary ophthalmologist (*n* = 2), a resident in training (*n* = 1), or a veterinarian in the national specialty training program for ophthalmology (*n* = 3). The ophthalmic examination included the menace response, the dazzle reflex, the pupillary light reflex, slit-lamp biomicroscopy (Kowa SL-17; Kowa, Tokyo, Japan), rebound tonometry (TonoVet, iCare, Vantaa, Finland), indirect ophthalmoscopy (Video Omega 2C; HEINE Optotechnik GmbH & Co. KG, Gilching, Germany), and, in some cases, gonioscopy (Supplementary Table 1).

Cats with a definitive histopathological diagnosis of FDIM or iris melanosis were included in the study. Eyes where the lesion was discovered only on post-enucleation histopathology, and no complete preoperative examination of the iris/anterior chamber was available, were excluded. The study population comprised cats that underwent enucleation following a clinical diagnosis or suspicion of FDIM, as well as cats with iris pigmentation that did not yet exhibit definitive clinical signs of FDIM but whose eyes were enucleated early at the owners’ request. The following data were collected for each case: breed, sex, age, days between the first examination and enucleation, days between suspicion of FDIM and enucleation, and reason for enucleation (veterinarian’s recommendation or owner’s decision). The presence or absence (binary assessment) of the following clinical parameters was recorded: iris thickening, dyscoria, reduced pupil motility, surface irregularities of the anterior iris surface, intraocular pressure (IOP), presence of glaucoma determined by clinical signs (e.g., buphthalmos, conjunctival or episcleral hyperemia, corneal edema, reduced pupillary light reflex or mydriasis, exposure keratitis, retinal degeneration, optic nerve cupping and IOP > 20 mmHg), occurrence of iris cysts and pigment dispersion (anterior chamber, anterior lens capsule, and iridocorneal angle). This binary approach is intrinsic to the nature of clinical ophthalmic examination in such cases, with the focus being on the identifiable presence of morphological markers as opposed to subjective grading. Histopathological parameters: melanoma, iris melanosis, progression to melanoma, invasion of the iris and trabecular meshwork, scleral invasion, extrascleral invasion, vascular invasion, choroidal invasion, necrosis, and closure of the iridocorneal angle were recorded for each globe.

### 2.1 Statistical analysis

The statistical analysis of the data was performed by Novustat GmbH (Wollerau, Switzerland). In order to analyze the potential factors influencing the occurrence of FDIM, a univariate analysis was performed on a group of biological and clinical predictors. To simplify the statistical analysis, castrated males and intact males were categorized together as “male,” while castrated females and intact females were grouped as “female.” Similarly, the histopathological diagnosis of progression to melanoma was grouped with the melanoma group. Each predictor was evaluated based on its log odds (logits) and the corresponding odds ratios (OR) for the diagnosis of FDIM. In a second step, a multivariate analysis was performed on the most influential parameters, using multiple logistic regressions. Given the small sample size of the non-FDIM cohort (*n* = 9), which limits the power of traditional confirmatory modeling, we adopted a refined exploratory multivariable logistic regression approach. This exploratory strategy was designed to identify clinically relevant trends while minimizing the risk of overfitting and ensuring model stability. To reduce the number of parameters to be estimated relative to the number of observed cases, the risk factors for pigment dispersion, previously recorded separately for the anterior chamber (AC) and anterior lens capsule (ALC), were combined into a single higher-level predictor indicating the presence of at least one of these forms. To further stabilize model estimation and minimize the inflation of odds ratios (ORs) and standard errors (SEs), the continuous predictors age (years) and intraocular pressure (IOP) were z-standardized prior to analysis. This refined exploratory model included *n* = 44 cats with *k* = 7 predictors (Supplementary material Data Sheet 1), providing a more robust basis for identifying clinically relevant trends within the study population. Missing data were handled using listwise deletion; specifically, cases with missing values for any clinical predictor included in a given model were excluded from that specific analysis. This explains the variation in sample sizes between the models (e.g., *n* = 44 for the multivariate analysis vs. *n* = 67 total), as only cats with complete data for all selected variables were included to ensure a consistent basis for the multivariate estimations. It is important to note that probabilities can only be calculated for a fixed value of the logarithmic probability (logit). This is due to the linear nature of the model in the logarithmic probabilities, while the probability of the relevant event (in this case, FDIM) is exponential-multiplicative. To allow for a deeper understanding of the effects investigated, the logits are supplemented by the corresponding ORs together with their 95% confidence intervals. This approach was used to clarify the effect in question, or their specific *p*-values are reported to assess their significance.

## 3 Results

### 3.1 Animals

A total of 67 cats met the inclusion criteria. The mean age ± standard deviation (SD) was 10.16 ± 3.69 years and ranged from 2 to 17 years. The data set included *n* = 27 (40.3%) female and *n* = 40 (59.7%) male animals. The domestic shorthair (DSH) was the most common breed, accounting for 42/67 (62.69%) of all cats. Other common breeds were British Shorthair (BSH) 13/67 (19.4%), DSH mix 4/67 (5.97%), Norwegian Forest Cat (NFC) mix 2/67 (2.99%), BSH mix, NFC, Persian, Selkirk Rex, Siberian Forest Cat mix, and Siamese mix were each represented once (1.49%) in the dataset. Compared to the breed distribution in the ophthalmology practice during the same period, the data set showed a significantly higher proportion of DSH cats (42.71% vs. 62.69%, *p* = 0.003).

### 3.2 Univariate analysis

The histopathological evaluation diagnosed FDIM in 58/67 (86.57%) cases and iris melanosis in 9/67 (13.43%) cases.

#### 3.2.1 Age

**Figure 2:**
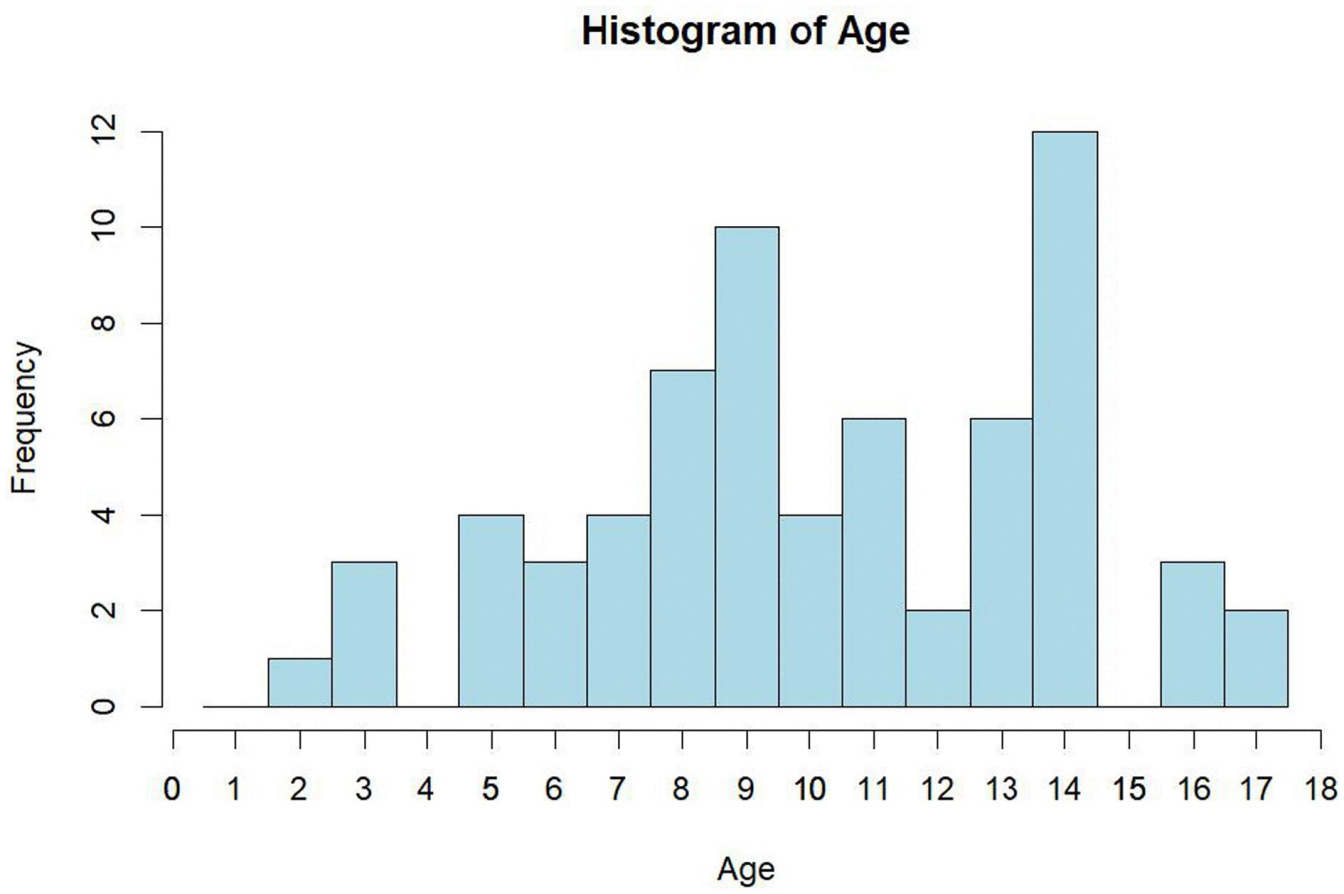
Age distribution of enrolled cats (*n* = 67). Bars show 1-year bins. The study population’s mean age was 10.16 ± 3.69 years (range 2–17), with the highest frequencies between 8 and 14 years.

The average age of cats in the group without FDIM was 7.33 ± 3.67 years, while the average age of cats in the FDIM group was 10.6 ± 3.52 years. The distribution of cats without FDIM shows a right-skewed distribution, indicating that the majority of cats were relatively young. The distribution of cats with FDIM is normally distributed around its mean. With each additional year of life, the probability of FDIM diagnosis increased by a factor of 1.3 (*p* = 0.02) (Figure 2).

#### 3.2.2 Sex

Female cats were more likely to be diagnosed with FDIM (88.89% vs. 85%). The probability of FDIM in female cats is increased by a factor of 1.41 (95% CI = 0.32–6.21, *p* = 0.65).

#### 3.2.3 Days from initial examination to enucleation, days from clinically suspected FDIM to enucleation

**Figure 3:**
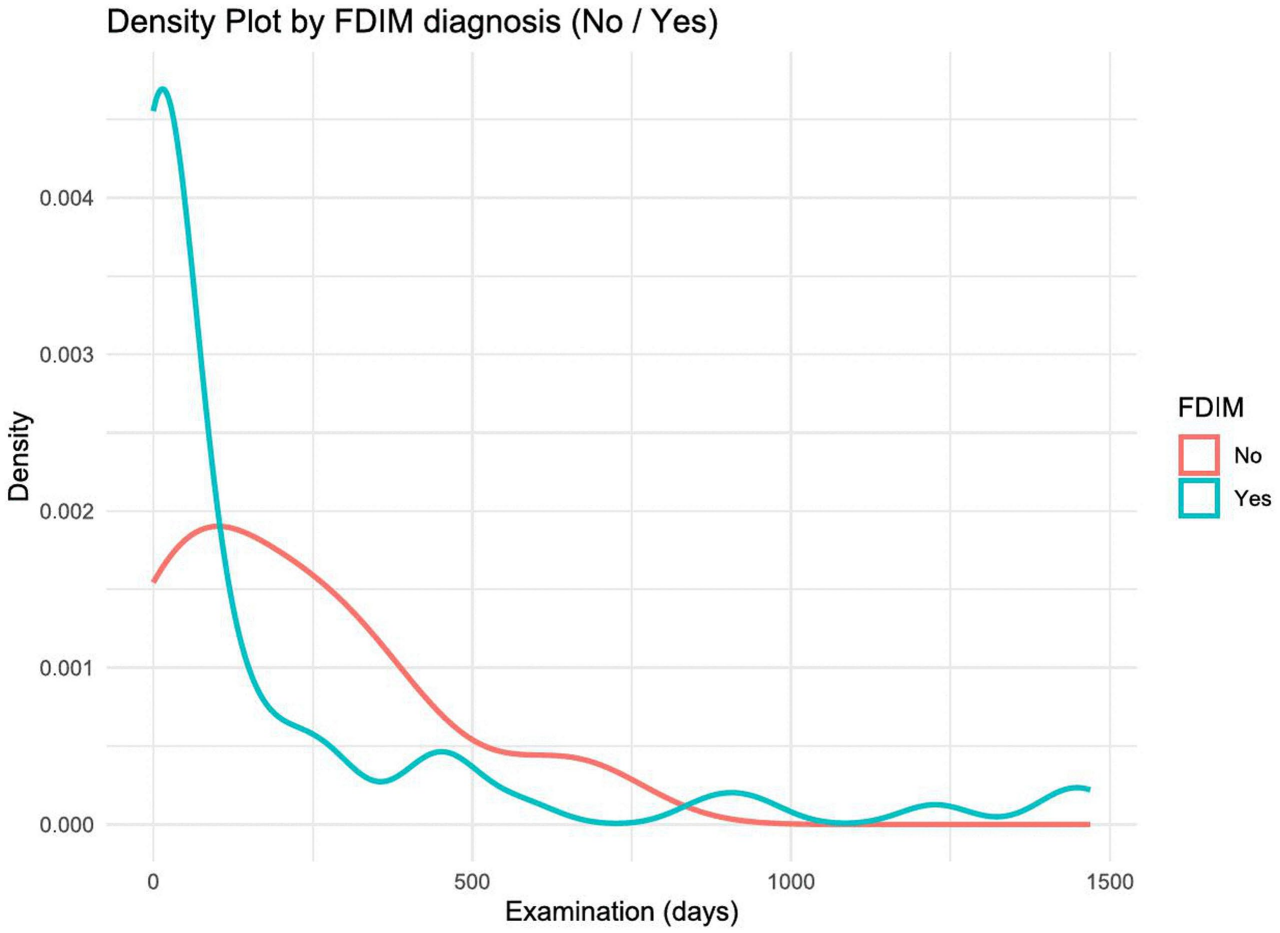
Density plot showing the distribution of time (in days) between first examination and enucleation, broken down by FDIM diagnosis (yes vs. no). The *x*-axis indicates the duration in days, while the *y*-axis represents the estimated probability density. Cats diagnosed with FDIM (blue line) have a more right-skewed distribution, with a higher density of shorter examination intervals compared to non-melanoma cases (red line).

The average number of days ± SD from initial examination to enucleation and from suspected FDIM to enucleation was 189.52 ± 332.62 and 38.97 ± 72.78, respectively. With each additional day between the initial examination and enucleation, the probability of melanoma decreased by a factor of 0.999 (*p* = 0.76) (Figure 3) With each additional day between suspicion of FDIM and enucleation, the probability of a diagnosis of FDIM decreased by a factor of 0.997 (*p* = 0.45).

#### 3.2.4 Reason for enucleation

Fifty-eight of 67 cats underwent enucleation on the recommendation of a veterinarian due to suspected FDIM. Nine of 67 cats underwent enucleation at the request of the owner. Although clinical changes were present, they were considered compatible with continued, close surveillance at that time. Cats whose eyes were removed on the recommendation of the veterinarian were diagnosed with FDIM 8.48 times more frequently (*p* < 0.05) than cats whose eyes were removed at the request of the owner. Cats whose eyes were removed at the owner’s request had an OR of 0.11 (*p* < 0.01) for a FDIM diagnosis.

#### 3.2.5 Iris thickening

**Figure 4:**
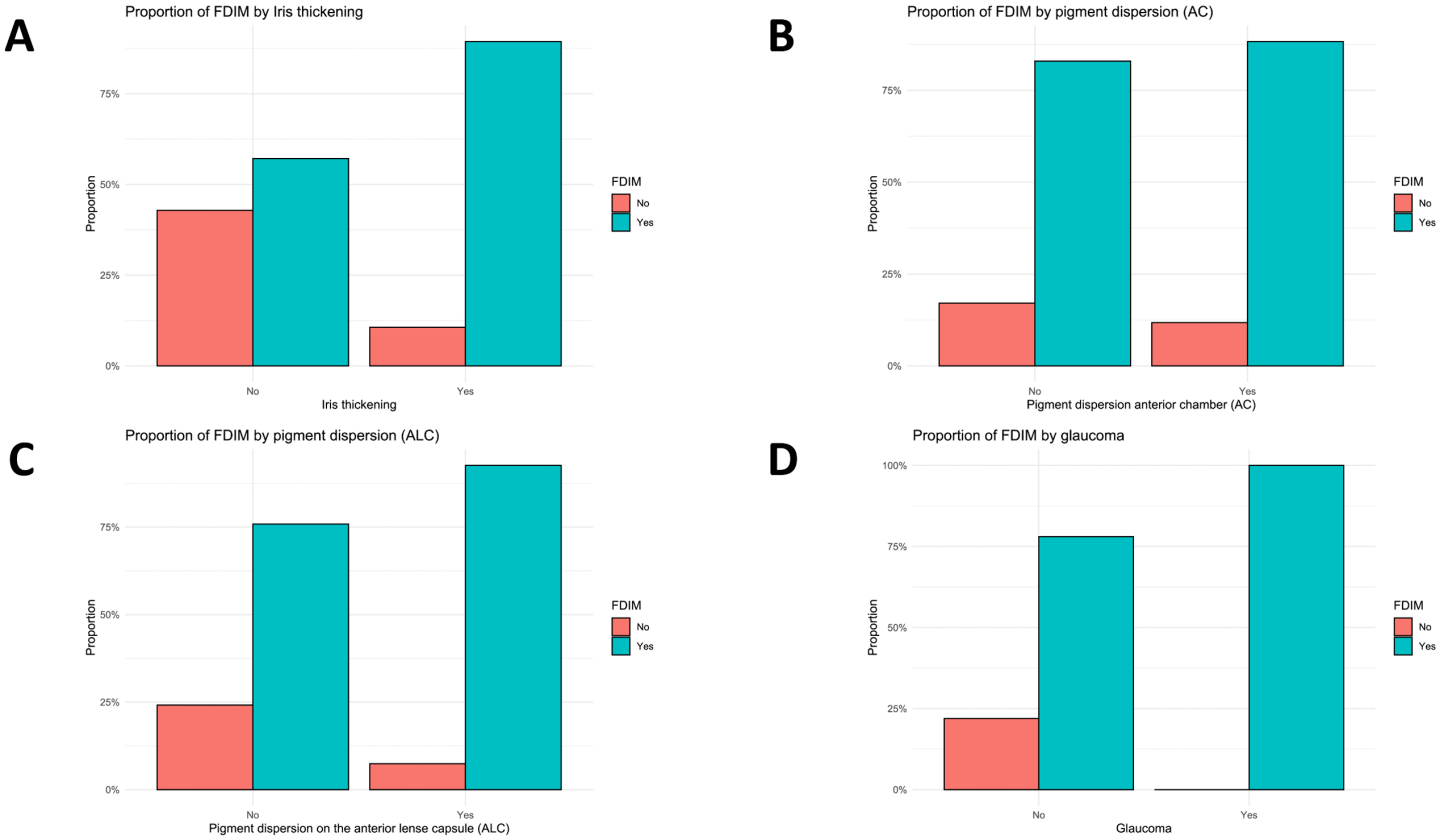
Proportion of cats diagnosed with FDIM in relation to **(A)** Iris thickening, **(B)** Pigment dispersion in the anterior chamber, and **(C)** Pigment dispersion on the anterior lens capsule. In each graph, ‘No’ indicates the absence and ‘Yes’ indicates the presence of the respective clinical symptom. A higher proportion of FDIM diagnoses was observed in cases where the clinical symptom was present, suggesting a possible association with malignant transformation. **(D)** Glaucoma: Proportion of FDIM diagnoses in eyes with versus without glaucoma. All glaucomatous eyes were diagnosed with FDIM, indicating a very strong association; in this subgroup, the absence of non-FDIM cases results in complete separation.

Cats with iris thickening (*n* = 47/54) had a higher probability of FDIM. They had a 6.3-fold higher probability (95% CI = 1.08–36.65, *p* = 0.058) compared to the baseline value without iris thickening (Figure 4A).

#### 3.2.6 Dyscoria

Cats with dyscoria (*n* = 11/56) had a lower probability of FDIM. In this case, the probability was 0.56 times (95% CI = 0.094–3.38, *p* = 0.61) lower than without dyscoria.

#### 3.2.7 Pigment dispersion

Cats with pigment dispersion in the anterior chamber (AC) (*n* = 17/58) had a higher probability of FDIM. They had a 1.54 times higher probability (95% CI = 0.29–8.33, p = 0.61) compared to the baseline value without pigment dispersion AC (Figure 4B).

Cats with pigment dispersion on the anterior lens capsule (ALC) (*n* = 27/56) had a higher probability of FDIM. They had a 3.98 times (95% CI = 0.75–21.18, *p* = 0.09) higher probability compared to the baseline value without pigment dispersion ALC (Figure 4C).

Cats with pigment dispersion in the iridocorneal angle (ICA) (*n* = 9/24) had a higher probability of FDIM. They had a 1.27 times (95% CI = 0.18–8.89, *p* = 0.81) higher probability compared to the baseline value without pigment dispersion.

#### 3.2.8 Intraocular pressure

**Figure 5:**
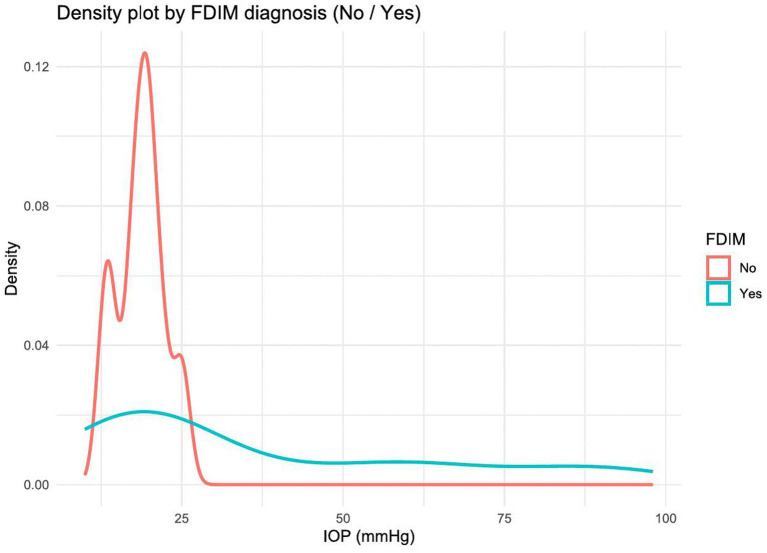
Density plot showing the distribution of intraocular pressure (IOP, in mm Hg) in cats with and without FDIM. Cats diagnosed with FDIM (blue line) showed a broader and right-shifted distribution of IOP values, including significantly elevated pressures, while non-melanoma cases (red line) showed a narrower distribution with peak values in the normal IOP range.

On average, cats in the group with iris melanosis had lower intraocular pressure (18.67 ± 3.74 mmHg) than cats in the FDIM group (37.84 ± 27.29 mmHg). The distribution of cats with and without FDIM is skewed to the right. However, cats without FDIM had a lower mean value and a significantly lower standard deviation, resulting in a more stepped distribution than cats with FDIM, which are distributed more widely around their respective mean values. With each additional mmHg, the probability of FDIM diagnosis increased by a factor of 1.077 (*p* = 0.12) (Figure 5).

#### 3.2.9 Glaucoma

Cats with glaucoma (*n* = 24/65) had an infinitely higher risk (*p* < 0.05) compared to the baseline value without glaucoma. There were no cats with glaucoma and a histopathological diagnosis of iris melanosis in the data collection (Figure 4D).

#### 3.2.10 Iris cysts

There were no cats with iris cysts in the dataset.

#### 3.2.11 Accuracy of the clinical diagnosis of FDIM or iris melanosis

Of the 58 enucleated eyes due to suspected FDIM by a veterinarian, 53 were FDIM, and 5 were iris melanosis. Of the 9 enucleated eyes due to the owner’s decision for an early enucleation, 5 were FDIM, and 4 were iris melanosis. The clinical diagnosis of FDIM or iris melanosis was correct in 85.07% of all cats. The correct diagnosis of FDIM is made on the basis of cats that were enucleated due to suspected FDIM and had a histopathological diagnosis of FDIM. Cases with a correct diagnosis of iris melanosis consist of cats that were enucleated at the owner’s request and received a histopathological diagnosis of iris melanosis. FDIM was correctly diagnosed in 53 of 58 cats, corresponding to a sensitivity of 91.38%. In four of nine cats, iris melanosis was correctly diagnosed, corresponding to a specificity of 44.44% (Table 1).

**Table 1:** Contingency table comparing preoperative clinical diagnosis with histopathological diagnosis in all included cases (*n* = 67).

Clinical diagnosis	Histopathology: FDIM	Histopathology: Iris melanosis
Clinical diagnosis: FDIM suspected	53 (True positive)	5 (False positive)
Clinical diagnosis: FDIM not yet suspected	5 (False negative)	4 (True negative)

FDIM, feline diffuse iris melanoma.

### 3.3 Multivariate analysis

**Figure 6:**
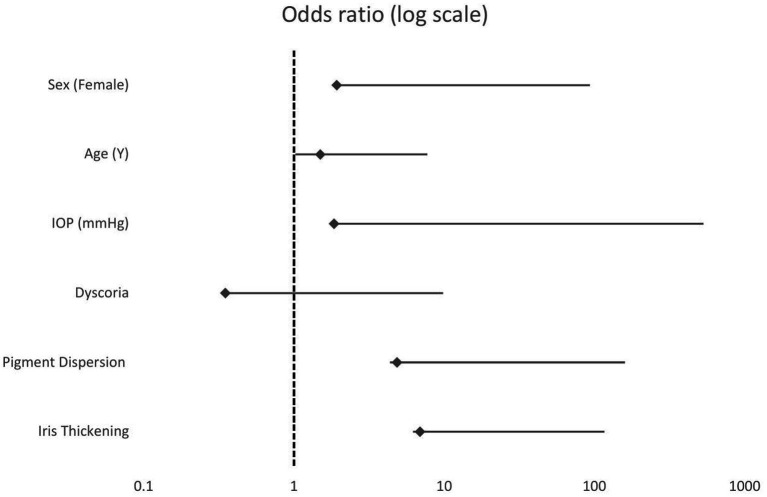
Forest plot illustrating the odds ratios and corresponding 95% confidence intervals (CI) derived from a multivariate logistic regression analysis based on data from 44 cats with the most complete clinical data for all predictor variables evaluated. Each row on the *y*-axis represents a clinical predictor that was evaluated for its association with the diagnosis of FDIM. The diamond-shaped markers indicate the point estimates of the ORs, while the horizontal lines show the respective 95% CI. The *x*-axis is logarithmically scaled. The dashed vertical line at OR = 1 marks the threshold for no association; values to the right indicate an increased likelihood, values to the left indicate a decreased likelihood of FDIM.

Iris thickening was strongly associated with the diagnosis of FDIM (OR = 6.91, 95% CI 0.6–108.01, *p* = 0.13). Other factors such as pigment dispersion (present vs. absent) (OR = 4.87, 95% CI = 0.44–152.70, *p* = 0.25), female sex (OR = 1.93, 95% CI = 0.22,21.21, *p* = 0.55), IOP (per +1 SD IOP: OR = 1.85, 95% CI = 0.14–523.54, *p* = 0.74), age (per +1 SD age: OR = 1.5, 95% CI = 0.46–6.11, *p* = 0.52) also showed increased odds ratios. No relevant associations were found for dyscoria (OR = 0.46, 95% CI = 0.02–9.37, *p* = 0.46). (Figure 6) The results of the univariate and explorative multivariate logistic regression analyses are summarised in Table 2. The complete model can be found in the Supplementary material Data Sheet 1.

**Table 2:** Odds ratios (OR), 95% confidence intervals (CI), and *p*-values for clinical parameters associated with the diagnosis of FDIM.

Clinical parameter	OR (univariate)	95% CI (univariate)	*p*-value (univariate)	OR (multivariate)	95% CI (multivariate)	*p*-value (multivariate)
Iris thickening	6.3	1.08–36.65	0.058	6.91	0.60–152.70	0.13
Pigment dispersion (ALC)	3.98	0.75–21.18	0.09	4.87	0.44–152.70	0.25
Pigment dispersion (AC)	1.54	0.29–8.33	0.61	4.87	0.44–152.70	0.25
Sex (female)	1.41	0.32–6.21	0.65	1.93	0.22–21.21	0.55
IOP (per mmHg)	1.077	-	0.12	1.85	0.14–523.54	0.74
Age (per year)	1.3	-	0.02	1.5	0.46–6.11	0.52
Dyscoria	0.56	0.094–3.38	0.61	0.35	0.02–9.37	0.46

Univariate and multivariate logistic regression analyses were performed.

CI, confidence interval; AC, anterior chamber; ALC, anterior lens capsule; IOP, intraocular pressure.

#### 3.3.1 Count of clinical findings

The number of clinical findings has been shown to have a positive, albeit insignificant, effect on the occurrence of FDIM, with a logit of approximately +0.36 (*p* = 0.36). With every additional clinical finding (+1), the chance of FDIM increased by a factor of 1.44 (95% CI 0.67–3.08).

### 3.4 Histopathological parameters

The histopathological parameters were not further examined in this study.

## 4 Discussion

This retrospective study aimed to evaluate the accuracy of diagnosing FDIM based solely on clinical parameters and to identify the clinical signs that are the strongest predictors of the disease. Our results showed that clinical assessment alone had a diagnostic accuracy of 85.07%. It had high sensitivity (91.38%), but relatively low specificity (44.44%). The high sensitivity may be attributable to the pronounced clinical features that are typically associated with advanced FDIM cases, such as iris thickening and pigment dispersion. These features facilitate accurate identification. In addition, the referral of advanced cases to our specialised ophthalmology practice, in addition to regular follow-up examinations for initially unremarkable or unclear cases, may have further increased the sensitivity. Regular ophthalmological follow-up examinations enable clinicians to detect subtle changes in clinical signs over time, thereby improving the identification of early malignant transformation. The authors recommend the use of photos for aiding the clinical decision-making.

The study found a comparatively low specificity, suggesting that it is a significant challenge to distinguish malignant FDIM from benign iris melanosis based on clinical features alone. This low specificity may be due to a significant overlap in the clinical presentations of these two conditions in the early stages, making differentiation without additional diagnostics difficult. In addition, the lack of advanced diagnostic methods that are routinely used in human ophthalmology (e.g., optical coherence tomography (19)) likely contributed to the relatively high false-positive rate.

Clinicians may also have taken a cautious approach to malignancy and recommended enucleation even in cases of high diagnostic uncertainty in order to reduce the risk of metastasis (5, 6), thereby reducing specificity. Univariate analysis revealed a significant association between age and FDIM, with the probability of malignancy increasing by a factor of 1.3 with each additional year of life. Studies show that FDIM often affects older cats, which is consistent with known epidemiological data and confirms its use as a clinical indicator (1, 2, 5).

Another finding was the association between female sex and FDIM (multivariate OR = 1.93, 95% CI = 0.22, 21.21, *p* = 0.55), a result that warrants further investigation due to its potential clinical significance. The available literature provides no evidence of a gender-specific predisposition (20). Further studies with larger cohorts could clarify whether this is a biological predisposition or an artefact due to the limited sample size of the study.

Of the clinical parameters examined, iris thickening showed the strongest association with a histopathological diagnosis of FDIM, with the highest odds ratio observed in both univariate and multivariate analyses (OR = 6.3; 95% CI = 1.08–36.65, *p* = 0.058; OR = 6.91, 95% CI 0.6–108.01, *p* = 0.13, respectively). This underscores its diagnostic significance as an important morphological indicator of malignancy.

Pigment dispersion in the anterior chamber or on the anterior lens capsule also proved to be a highly predictive feature (multivariate OR = 4.87, 95% CI = 0.44–152.70, *p* = 0.25), indicating pigment detachment from the iris and possibly pointing to tumour invasion or progression. Similarly, pigment deposition on the anterior lens capsule was associated with a significantly increased likelihood of FDIM (univariate OR = 3.98, 95% CI = 0.75–21.18, *p* = 0.09). Since posterior synechia could also lead to pigment deposition on the anterior lens capsule, this parameter should be interpreted with caution.

In the subgroup analysis for glaucoma, complete separation (21) occurred because every cat in this group was histopathologically diagnosed with FDIM. The absence of outcome variation, specifically the lack of cats with both glaucoma and iris melanosis, prevents the logistic regression model from converging on a finite maximum likelihood estimate. This causes the odds ratio to approach infinity and results in model instability. Whilst this complete separation precludes a stable numerical estimation of the odds ratio, it reflects the clinical reality that glaucoma was exclusively associated with FDIM in the present cohort. This highlights its role as a critical indicator of advanced malignancy, despite the inherent statistical limitations of the model.

These results underscore the importance of a detailed examination of the anterior segment of the eye during an ophthalmological examination. The simultaneous presence of iris thickening and pigment dispersion, especially within the anterior chamber, should raise a strong suspicion of FDIM and justify enucleation, even in the absence of glaucoma or obvious tumour nodules.

In human ophthalmology, the clinical diagnosis of iris melanoma is based on the size of the tumor, morphological characteristics, and proven growth patterns over a certain period of time (22). In addition to clinical examination, additional diagnostic methods such as fine needle aspiration (23) and high-resolution ultrasound imaging (24) are routinely used to distinguish iris melanoma from benign lesions such as iris nevi. Conversely, there is currently a lack of widely available complementary diagnostic tools in veterinary ophthalmology. Apart from histopathological confirmation after enucleation, iris biopsy is the only minimally invasive technique available. This method was first described by Koch et al. (17) and further evaluated by Featherstone et al. (16). Both studies reported that the procedure was associated with only minor complications (16, 17). In the study by Featherstone et al., FDIM was histopathologically confirmed after enucleation in two of seven cases in which an iris biopsy had been performed previously, providing preliminary validation of the technique (16).

In a recent study, Beetz et al. (18) investigated the diagnostic concordance between iris biopsy samples ex vivo and the corresponding globe. The samples were assessed by eight different pathologists, with an average agreement rate of 81.6%. It is noteworthy that this value is slightly below the diagnostic accuracy of 85.07% determined in our study based solely on clinical assessment. Beetz et al. (18) identified sample quality and disease stage as decisive factors influencing diagnostic agreement. In particular, it was found that poor-quality biopsy material and FDIM in the early stages are associated with higher rates of diagnostic discrepancies. The clinical features most frequently documented in the study by Beetz et al. (18) that preceded enucleation, such as iris thickening and pigment dispersion, correspond to the clinical predictors that showed high odds ratios in our analysis. However, a significant discrepancy was found in the prevalence of dyscoria: this occurred in 76.47% of cases in the Beetz cohort, but only in 19.64% of cases in the present cohort. The relatively low prevalence of dyscoria in our cohort compared to other studies (2, 16, 18, 25) may be due to earlier referral, different documentation standards, or overly conservative clinical interpretation of pupil shape in our cases.

In a pilot study, Komatsu et al. used optical coherence tomography to assess pigmented iris lesions in cats (26). In contrast to FDIM, hyperreflective lines with greater intensity than the surrounding iris stroma were observed in the areas affected by iris melanosis. Furthermore, cases of early-stage FDIM showed a 1.3-fold increase in iris thickness compared to normal eyes (26). These results are consistent with the findings of the present study, in which iris thickening was identified as a significant clinical predictor in both univariate and multivariate analyses.

When we compared our results with prediction models from human ophthalmology, several parallels emerged. Singh et al. (15) identified the following characteristics as the strongest clinical predictors of iris melanoma: increased tumour height (OR 3.35); larger base diameter (OR 1.64); pupil distortion, such as ectropion uveae or corectopia (OR 2.55); peripheral extension with involvement of the iridocorneal angle or iris root (OR 2.83); pigment dispersion (OR 1.12) and tumor vascularity (OR 6.79). These parameters overlap conceptually with those identified in our study. In particular, iris thickening, which may be analogous to increased tumour height, showed the highest predictive value in our dataset, with an odds ratio of 6.3 in the univariate analysis and 4.87 in the multivariate analysis. Similarly, pigment dispersion in the anterior chamber was associated with an increased likelihood of FDIM, with odds ratios of 1.54 (univariate). Pigment dispersion on the anterior lens capsule also showed elevated odds (OR 3.98 in univariate analysis).

Fuchs et al. proposed a new grading system for progressive pigmented iris lesions in cats, ranging from iris nevi to FDIM grades one to five (14). This system takes into account the degree of iris involvement, iris thickening, the presence of uveitis, inflammatory and pigment cells, dyscoria, and secondary glaucoma (14). Our data support this model and show that iris thickening and pigment dispersion in the anterior chamber and on the anterior lens capsule, as well as glaucoma, were associated with the highest odds ratios for a diagnosis of FDIM. However, the univariate model for glaucoma represents a limitation of our study. In this subgroup analysis, all cats diagnosed with glaucoma were also diagnosed with FDIM, resulting in a complete separation of the outcome variables. Consequently, the odds ratio approaches infinity, suggesting that glaucoma perfectly predicts FDIM in this sample. Although this suggests a strong correlation, the statistical model cannot reliably estimate the effect due to the lack of variation in the results. Especially since no cats with glaucoma and iris nevi were included in the dataset.

The intervals from initial examination to enucleation and from clinical suspicion to enucleation showed odds ORs slightly below 1 (0.999 and 0.997, respectively) with wide dispersion and no statistical significance. Rather than suggesting a protective effect, this is more likely to be a reflection of confounding by indication and referral dynamics. Eyes that were judged to be more suspicious were enucleated earlier, whereas lesions that were considered to be benign or equivocal underwent surveillance, resulting in a longer interval among non-FDIM cases. Owner preference and scheduling constraints may have had a role to play in this outcome. It is therefore imperative that these temporal variables are interpreted as markers of clinical decision-making, rather than disease biology.

This study has several limitations that need to be taken into account. The retrospective design carries a potential risk of selection and information bias, particularly given that the study was conducted in a single ophthalmology practice, which may have led to an overrepresentation of advanced or atypical cases. The relatively small number of cats with histologically confirmed iris melanosis limited the statistical significance of the findings, particularly in the multivariate model. Furthermore, not all clinical variables were documented in every case, which reduced the data set available for multivariate analysis and may have introduced bias due to missing data. Although all ophthalmic examinations were performed by experienced veterinary clinicians, a formal assessment of interobserver variability was not conducted due to the retrospective study design. Consequently, variability in the evaluation of subjective clinical parameters, such as dyscoria or iris thickening, cannot be excluded. Finally, the study focused on diagnostic accuracy and did not evaluate the prognostic implications of the clinical findings.

It is important to note that the small sample size of the iris melanosis group (*n* = 9), which contributed to the statistical instability of the multivariate model, is a direct result of high clinical diagnostic accuracy. From a clinical and ethical standpoint, the minimisation of false positive enucleations of eyes with benign lesions is considered a primary objective. The fact that 91.38% of eyes recommended for enucleation by a veterinarian were confirmed as FDIM demonstrates that clinical signs such as iris thickening and pigment dispersion are highly effective filters in practice. Whilst the distribution is skewed, thus limiting statistical power, this nevertheless highlights the reliability of the clinical assessment in protecting cats from unnecessary surgery.

## 5 Conclusion

Our results demonstrate that a thorough clinical assessment achieves a diagnostic accuracy of 85.07%, which is comparable to the reported concordance rates for invasive methods such as iris biopsy (81.6%). This suggests that clinical evaluation by experienced ophthalmologists is a highly reliable tool for treatment decisions, performing on par with more invasive diagnostic procedures. The results of this study underscore the importance of thorough clinical examination and regular re-evaluation and indicate that further research into minimally invasive diagnostic techniques in veterinary ophthalmology is needed. The reported assessment regarding breed and sex requires further investigation.

## Data Availability

The original contributions presented in the study are included in the article/Supplementary material, further inquiries can be directed to the corresponding author/s.

## References

[1] Dubielzig RR. "Tumors of the eye". In: Tumors in Domestic Animals (2016). p. 892–922.

[2] Kayes D Blacklock B. Feline uveal melanoma review: our current understanding and recent research advances. Veterinary Sciences. (2022) 9:46. doi: 10.3390/vetsci9020046, 35202299 PMC8877522

[3] Shields JA Sanborn GE Augsburger JJ. The differential diagnosis of malignant melanoma of the iris. A clinical study of 200 patients. Ophthalmology. (1983) 90:716–20. doi: 10.1016/s0161-6420(83)34500-0, 6888862

[4] Fragola JA Dubielzig RR Bentley E Teixeira LBC. Iridociliary cysts masquerading as neoplasia in cats: a morphologic review of 14 cases. Vet Ophthalmol. (2018) 21:125–31. doi: 10.1111/vop.12484, 28685998

[5] Kalishman Chappell Flood Dubielzig. A matched observational study of survival in cats with enucleation due to diffuse iris melanoma. Vet Ophthalmol. (1998) 1:25–9. 11397206 10.1046/j.1463-5224.1998.00006.x

[6] Wiggans KT Reilly CM Kass PH Maggs DJ. Histologic and immunohistochemical predictors of clinical behavior for feline diffuse iris melanoma. Vet Ophthalmol. (2016) 19:44–55. 26805705 10.1111/vop.12344

[7] Rushton JG Ertl R Klein D Tichy A Nell B. Circulating cell-free DNA does not harbour a diagnostic benefit in cats with feline diffuse iris melanomas. J Feline Med Surg. (2019) 21:124–32. doi: 10.1177/1098612X18762017, 29529957 PMC10814613

[8] Rushton JG Ertl R Klein D Nell B. Mutation analysis and gene expression profiling of ocular melanomas in cats. Vet Comp Oncol. (2017) 15:1403–16. doi: 10.1111/vco.12285, 28074614

[9] Rushton JG Korb M Kummer S Reichart U Fuchs-Baumgartinger A Tichy A . Protein expression of KIT, BRAF, GNA11, GNAQ and RASSF1 in feline diffuse iris melanomas. Vet J. (2019) 249:33–40. doi: 10.1016/j.tvjl.2019.04.008, 31239162

[10] Duncan D Peiffer R. Morphology and prognostic indicators of anterior uveal melanomas in cats. Prog Vet Comp Ophthalmol. (1991) 1:25–32.

[11] Patnaik AK Mooney S. Feline melanoma: a comparative study of ocular, oral, and dermal neoplasms. Vet Pathol. (1988) 25:105–12. doi: 10.1177/030098588802500201, 3363787

[12] Dubielzig RR Ketring KL McLellan GJ Albert DM. (2010). Veterinary Ocular Pathology: a Comparative Review.

[13] Acland GM McLean IW Aguirre GD Trucksa R. Diffuse iris melanoma in cats. J Am Vet Med Assoc. (1980) 176:52–6. doi: 10.2460/javma.1980.176.01.52, 7351385

[14] Fuchs AA Giuliano EA English R Nadelstein B. Diode laser ablation of progressive pigmented iris lesions in 317 cats (356 eyes) appears overall safe and effective in decreasing progression of iris pigmentation. J Am Vet Med Assoc. (2024) 262:117–24. doi: 10.2460/javma.23.07.0387, 37758183

[15] Singh A Melendez-Moreno A Krohn J Zabor EC. Predictive model for iris melanoma. Br J Ophthalmol. (2024) 108:1598–604. doi: 10.1136/bjo-2023-324558, 38609162

[16] Featherstone HJ Scurrell EJ Rhodes M de Pinheiro Lacerda R. Iris biopsy to investigate feline iris hyperpigmentation. Vet Ophthalmol. (2020) 23:269–76. doi: 10.1111/vop.12718, 31733046

[17] Koch E Bouhanna L Goulle F. Iris Biopsy: a Useful tool in the Early Diagnosis of feline Diffuse iris melanoma Abstracts: Annual Scientific Meeting of the European College of Veterinary Ophthalmologists. Antwerp, Belgium: Veterinary Ophthalmology (2019). p. E1–E27.

[18] Beetz S Klopfleisch R Naranjo C Scurrell E Bushe J Lehmbecker A . Diagnostic value of iris biopsies for the assessment of iris pigment changes and diffuse iris melanoma (DIM) in cats: a descriptive histopathological study. Vet Pathol. (2025) 3009858251403170. 41474024 10.1177/03009858251403170 PMC13254134

[19] Patel DR Blair K Patel BC. Ocular Melanoma. Treasure Island, FL: StatPearls Publishing (2025). 31869111

[20] Lo Giudice A Porcellato I Giglia G Sforna M Lepri E Mandara MT . Exploring the epidemiology of melanocytic tumors in canine and feline populations: a comprehensive analysis of diagnostic records from a single pathology institution in Italy. Vet Sci. (2024) 11. doi: 10.3390/vetsci11090435, 39330814 PMC11436034

[21] Albert A Anderson JA. On the existence of maximum likelihood estimates in logistic regression models. Biometrika. (1984) 71:1–10. doi: 10.1093/biomet/71.1.1

[22] Cherkas E Kalafatis NE Marous MR Shields CL. Iris melanoma: review of clinical features, risks, management, and outcomes. Clin Dermatol. (2024) 42:62–70. doi: 10.1016/j.clindermatol.2023.10.009, 37865279

[23] Shields CL Manquez ME Ehya H Mashayekhi A Danzig CJ Shields JA. Fine-needle aspiration biopsy of iris tumors in 100 consecutive cases: technique and complications. Ophthalmology. (2006) 113:2080–6. doi: 10.1016/j.ophtha.2006.05.042, 17074566

[24] Conway RM Chew T Golchet P Desai K Lin S O’Brien J. Ultrasound biomicroscopy: role in diagnosis and management in 130 consecutive patients evaluated for anterior segment tumours. Br J Ophthalmol. (2005) 89:950–5. doi: 10.1136/bjo.2004.059535, 16024841 PMC1772796

[25] Jajou S. Uveal amelanotic melanoma in a ragdoll cat. Can Vet J. (2020) 61:645–7. 32675817 PMC7238484

[26] Komatsu H Akasaka M Morita M Usami K Inagaki M Kumashiro K . A pilot study to evaluate the usefulness of optical coherence tomography for staging iris pigmented lesions in cats. Vet Sci. (2024) 11:261. doi: 10.3390/vetsci11060261, 38922008 PMC11209344

