# Supplementary material for: Assessment of the diagnostic accuracy of clinical signs in feline diffuse iris melanoma
Source: Front Vet Sci. 2026 Mar 23;13:1795617. doi: 10.3389/fvets.2026.1795617 (PMC13051678; doi:10.3389/fvets.2026.1795617)

**Multivariate analysis**

| **Predictor** | **Logit (B)** | **S.E.** | **z** | **p** | **OR** | **95% CI (OR)** |
| --- | --- | --- | --- | --- | --- | --- |
| Intercept | 0.17 | 1.69 | 0.10 | 0.92 | 1.19 | [0.05; 90.20] |
| Sex (female) | 0.66 | 1.10 | 0.60 | 0.55 | 1.93 | [0.22; 21.21] |
| Age (years) | 0.41 | 0.63 | 0.65 | 0.52 | 1.50 | [0.46; 6.11] |
| IOP (mmHg) | 0.61 | 1.84 | 0.33 | 0.74 | 1.85 | [0.14; 523.54] |
| Dyscoria (Yes) | -1.05 | 1.42 | -0.74 | 0.46 | 0.35 | [0.02; 9.37] |
| Iris thickening (Yes) | 1.93 | 1.27 | 1.53 | 0.13 | 6.91 | [0.60; 108.01] |
| Pigment dispersion (Yes) | 1.58 | 1.38 | 1.15 | 0.25 | 4.87 | [0.44; 152.70] |

N = 44, LR χ² (6) = 9.69 (*p* = .14)

- Sex (female): OR = 1.93, 95% CI [0.22; 21.21], *p* = .551 → female cats show a 1.93-fold chance of FDIM compared to male cats. Positive but insignificant effect.
- Age (per +1 SD age): OR = 1.50, 95% CI [0.46; 6.11], *p* = .518 → a higher age of +1 SD is associated with a 1.50-fold chance of FDIM. Positive but insignificant effect.
- IOP (per +1 SD IOP): OR = 1.85, 95% CI [0.14; 523.54], *p* = .738 🡪 a higher IOP of +1 SD is associated with a 1.85-fold chance of FDIM. Positive but insignificant effect.
- Dyscoria (Yes vs No): OR = 0.35, 95% CI [0.02; 9.37], *p* = .459 → dyscoria is associated with a 0.35-fold chance of FDIM. Negative but insignificant effect.
- Iris thickening (Yes vs No): OR = 6.91, 95% CI [0.60; 108.01], *p* = .127 → iris thickening is associated with a 6.91-fold chance of Iris FDIM. Strongest positive Effect but still insignificant.
- Pigment dispersion (present vs absent): OR = 4.87, 95% CI [0.44; 152.70], *p* = .252 → Pigment Dispersion (AC or ALC) is associated with a 4.87-fold chance of Iris Melanoma. Second strongest positive effect but still insignificant.

| **Matrix of Classification** | | |
| --- | --- | --- |
| Predicted / Actual | No | Yes |
| No | 2 | 1 |
| Yes | 4 | 37 |

- Accuracy = 88.64%
- 86.64% of cats are classified correctly by the fitted logistic model for FDIM (No / Yes).
- from 6 cats without FDIM the model correctly detects 2 Cats 🡪 low specificity.
- from 38 cats with FDIM the model correctly detects 37 Cats 🡪 very high sensitivity.


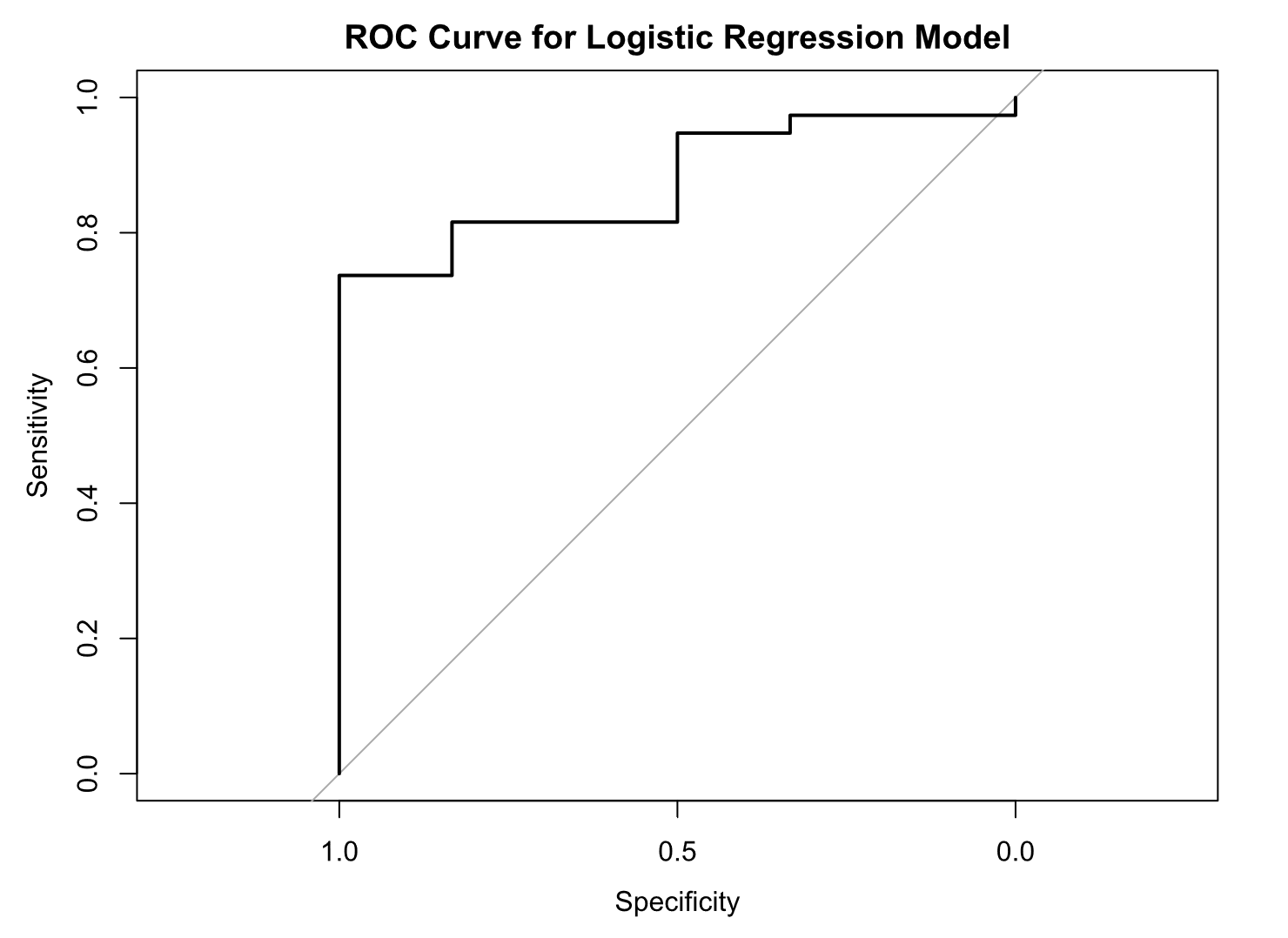

Supplement: Supplementary file 2 [file Data_Sheet_1.DOCX]
